# Associations between Parents’ Perceived Air Quality in Homes and Health among Children in Nanjing, China

**DOI:** 10.1371/journal.pone.0155742

**Published:** 2016-05-18

**Authors:** Hua Qian, Xiaohong Zheng, Min Zhang, Louise Weschler, Jan Sundell

**Affiliations:** 1 School of Energy and Environment, Southeast University, Nanjing, China; 2 Department of Building Science, Tsinghua University, Beijing, China; Telethon Institute for Child Health Research, AUSTRALIA

## Abstract

The increasing prevalence of respiratory diseases in Chinese children has focused attention on indoor environmental quality. We investigated associations between perceived air quality in domestic environments and children’s allergic diseases with a questionnaire survey study. A total of 4017 children aged 1–8 years old from 23 kindergartens in urban, suburban and industrial areas in Nanjing were randomly recruited for this study. Parents’ perceived odors, including stuffy odor, unpleasant odor, pungent odor, moldy odor, humid air and dry air were found to be associated with asthma, wheeze, dry cough and rhinitis (*P* < 0.05). Both perceived dry and humid air were found to be positively associated with dampness indices, and we present evidence that the sensation of dryness may not be due to the actual indoor relative humidity, but rather to indoor air irritants. Parents’ perception of odors and relative humidity may be indicators of environment pollutants, which are likely the real factors associated with children’s allergic diseases.

## Introduction

Prevalences of asthma and allergic diseases worldwide are increasing, and this is particularly the case in China where a dramatic rise in the prevalence of allergic diseases has been reported in the past two decades [1,2]. The indoor environment, especially in the home, likely has a significant role in inducing asthma and allergic diseases among children [3–11]. The present trend in asthma prevalence [1] parallels rapid growth in the Chinese economy, and the simultaneous rise in the market value of real estate [12]. Most modern apartments are more airtight than older apartment buildings, allowing pollutants generated indoors to accumulate [13]. New furniture and decoration release formaldehyde and other respiratory irritants [14–16]. Home refurbishment/decoration/remodeling activities in newly constructed or existing apartments have become major environmental health concerns in China [14,17] particularly with respect to young children [1].

Perceived air quality, i.e. perception of odors and the sensation of irritation, has been shown to be related to indoor pollutants, e.g. VOCs, formaldehyde and NO_x_ [18]. Perception of both odors and relative humidity may be influenced by ventilation [7]. Building dampness may lead to different types of exposures including mold odor produced by micro-organisms [19]. Because it is difficult to separate the effects of organic compounds, ventilation levels and micro-organisms, the combined effects of sensory irritants in humans can be estimated as additive for a first approximation [20].

There have been studies of associations between odor and humidity perception and human health outcomes in many countries. In 1993, the sensation of either dampness or dryness were both found to be indicators of SBS [21]. Adult perceptions of odors and either humid or dry air were found to be associated with SBS symptoms by Wang et al. in their Chongqing, China study [20]. Wang et al.’s study also showed that mold odor, an indicator of dampness, was a significant risk factor for asthma and all asthma and allergic symptoms among children in Chongqing [22]. Their finding is consistent with those of previous studies [23,24]. Perceived odors were also found to be related to asthma symptoms and dry cough among adults in old multi-family houses in Sweden [19].

There have been few previous studies of parents’ perceptions of odors and humidity as related to children’s diagnosed asthma, other allergic diseases and pneumonia. The objective of this study is to investigate associations between parents’ perceived indoor air quality (odors and relative humidity) and children’s health in the city of Nanjing. We also investigated associations between building characteristics of homes and parents’ perceptions of odors and relative humidity.

## Methods

### Ethics Statement

The Medical Research Ethics Committee of the School of Public Health, Fudan University approved our study and consent procedure (International Registered Number: IRB00002408&FWA00002399). Participants gave informed consent to respond to the questionnaire survey.

### Questionnaire

The present study is part of the cross-sectional study in China, “Phase 1 of CCHH (China children Home Health) study [1].” The CCHH questionnaire survey [1,25] was used to investigate health outcomes and home environmental exposure of Nanjing children less than 8 years old [1,25]. Medical history questions were taken from the ISAAC (International Study of Asthma and Allergies in Childhood) questionnaire, and questions about environmental factors were based on those in the DBH (Dampness in Building and Health) study conducted in Sweden [9], appropriately modified for Chinese culture, lifestyle, building structure and interior characteristics.

A total of 83 questions was divided into 6 sections: demographic information, health history for children and their family members, family life style, building and indoor environment characteristics, and diet. The questions about children's health, building characteristics and perceived air quality are shown in the S1 File.

### Study areas

This questionnaire study was carried out in Nanjing, the capital of Jiangsu Province, located in the Yangtze River Delta economic zone, the largest and one of most developed economic zones in China. The climate is hot and humid in summer and cold and humid in winter. Nanjing has a total of 8 urban districts, 2 suburban districts, 1 industrial district and 2 rural counties. All except the two rural counties were included in this study. One suburban district and one industrial district are located north of the Yangtze River and other districts are located south of the Yangtze River. The heavy machinery and chemical factories are concentrated in the industrial district.

### Study populations

The survey was conducted from December 2010 to March 2011 in Nanjing. Our study focused on children less than 8 years of age in kindergartens. Twenty-three (23) kindergartens were randomly recruited in the 11 districts of Nanjing, covering urban, suburban and industrial areas. Because children’s homes are generally within walking distance to the kindergarten in both urban and sub-urban areas, we used the kindergarten locations to denote the children’s home locations. We checked contact addresses in the questionnaires to validate this assumption.

Questionnaires were distributed by kindergarten teachers in each kindergarten to a total of 6461 children as homework for their parents. The parents filled in the questionnaire and the children submitted the completed questionnaires to their teachers. We collected the questionnaires and input their data into a database.

### Data analysis

The statistical analysis was conducted using SPSS for windows (SPSS Incorporated, SPSS Release 17.0). Initially, associations of parent’s perceived odors with gender, history of asthma or allergies, location of home, construction year, home floor area, and dampness indices were analyzed by Chi-square analysis. Then, Chi-square tests were used to crudely estimate associations between parents’ perceived odors and children’s health. Finally, odds ratios for children’s health associations with parent’s perceived odors were calculated by binary regression adjusted for children’s gender, age and family asthma or allergy history. Odds ratios were calculated with 95% confidence intervals (CI). *P*-value less than 0.05 indicated a statistically significant level.

An index score was created to estimate the severity of odor and humidity (O&H) exposure and the score was categorized and applied in logistic regression models to analyze associations between the categorized O&H score and children’s allergic diseases.

## Results

Questionnaires were distributed to 6461 children; 4017 were properly completed and returned, giving a response rate of 65.7%. The children were 51.2% male and 48.8% female; 1.1% of the children were less than 3 years old, 35.1% were 3–4 years old, 50.1% were 5–6 years old and 13.8% were 7–8 years old. 96.3% of the questionnaires were completed by parents, 3.1% by grandparents and 0.6% by other guardians. Fathers or other male guardians filled in 27.1% of the questionnaires.

Table 1 shows the proportions of perceived types of odor and the sensation of relative humidity in surveyed homes. Tobacco odor was the most commonly perceived weekly odor. The sensation of air dryness was the most commonly perceived sensation of indoor air reported by both male guardians and female guardians (weekly and sometimes). A higher proportion of female than male guardians reported stuffy and unpleasant odors, and perceived humid and dry air (*P* < 0.05). Higher proportions of guardians with a family history of asthma or allergies reported stuffy, unpleasant, pungent odors, as well as the sensation of both humid air and dry air (*P* < 0.05).

**Table 1:** Proportions of perceived odors and the sensation of humidity, stratified according to guardians’ gender and family history of asthma or allergies.

Category	Frequency	Total	Gender		*P*	History of asthma or allergies		*P*
			Male	Female		Yes	No	
Stuffy odor	weekly	2.6	2.0	2.9	**<0.001**	4.5	2.2	**<0.001**
	sometimes	37.0	29.5	39.7		44.3	35.2	
Unpleasant odor	weekly	1.3	1.1	1.3	**0.001**	1.7	1.1	**<0.001**
	sometimes	24.5	20.1	26.1		30.5	23.1	
Pungent odor	weekly	0.5	0.7	0.4	0.051	0.5	0.5	**0.016**
	sometimes	10.4	8.7	11.1		12.6	9.6	
Moldy odor	weekly	0.4	0.2	0.5	0.294	0.5	4.0	0.056
	sometimes	9.3	10.0	9.0		11.9	8.8	
Tobacco odor	weekly	6.1	4.6	6.7	0.055	6.9	5.9	0.466
	sometimes	28.8	28.9	28.7		27.3	29.1	
Humid air	weekly	1.1	1.0	1.1	**0.034**	1.7	1.0	**<0.001**
	sometimes	34.8	31.5	36.1		42.3	33.4	
Dry air	weekly	3.2	1.7	3.7	**<0.001**	5.1	2.8	**<0.001**
	sometimes	48.0	44.5	49.3		51.9	47.1	

The surveyed homes were 52.7% in urban, 38.9% in suburban and 8.3% in industrial areas. Proportions of perceived odors according to residence location are given in Table 2. Perceptions of unpleasant odor, pungent odor and tobacco odor in industrial areas was significantly higher than in urban and suburban areas (*P* < 0.05).

**Table 2:** Odors and humidity perceptions, stratified for dwelling locations of surveyed homes and construction year of the buildings.

		Location			Building construction time		
Category	Frequency	Urban	Suburban	Industrial	Before 1990	1991–2000	After 2001
Stuffy odor	weekly	2.7	2.6	1.9	2.6	2.7	2.4
	sometime	36.3	37.1	36.3	38.7	36.4	35.7
Unpleasant odor	weekly	1.0***	1.1***	3.2***	0.6	1.9	1.1
	sometime	25.7***	21.8***	27.3***	25.8	24.9	23.4
Pungent odor	weekly	0.4***	0.3***	1.9***	0.6	0.3	0.5
	sometime	10.3***	8.8***	17.0***	10.6	10.0	10.0
Moldy odor	weekly	0.5*	0.3*	0.3*	0.9***	0.5***	0.3***
	sometime	10.9*	7.2*	9.3*	13.8***	10.5***	7.0***
Tobacco odor	weekly	6.2*	5.8*	6.6*	9.2	5.8	5.3
	sometime	26.4*	31.2*	33.9*	28.3	27.0	29.7
Humid air	weekly	1.3*	0.9*	0.3*	1.8*	1.2*	0.7*
	sometime	36.8*	31.4*	36.5*	37.8*	35.5*	32.9*
Dry air	weekly	2.8	3.5	3.5	4.0	1.9	3.3
	sometime	47.6	48.0	47.9	45.9	50.9	46.9

* *P* < 0.05

*** *P* < 0.001.

The majority of homes was built after 2000 (53.7%); 26.6% were built between 1991 and 2000. Table 2 shows that moldy odor and the sensation of humid air were more common in older homes (*P* < 0.01).

The majority of homes (56.8%) had floor area greater than 75 m^2^. Significantly higher (*P* < 0.05) proportions reported perceived odors in smaller dwellings (< 75 m^2^) than in larger dwellings (> 75m^2^): unpleasant (28.1% versus 23.6%) and moldy (12.3% versus 8.0%) odors as well as of humidity (37.5% versus 34.1%). Detailed data are presented in S1 Table.

**Fig 1:**
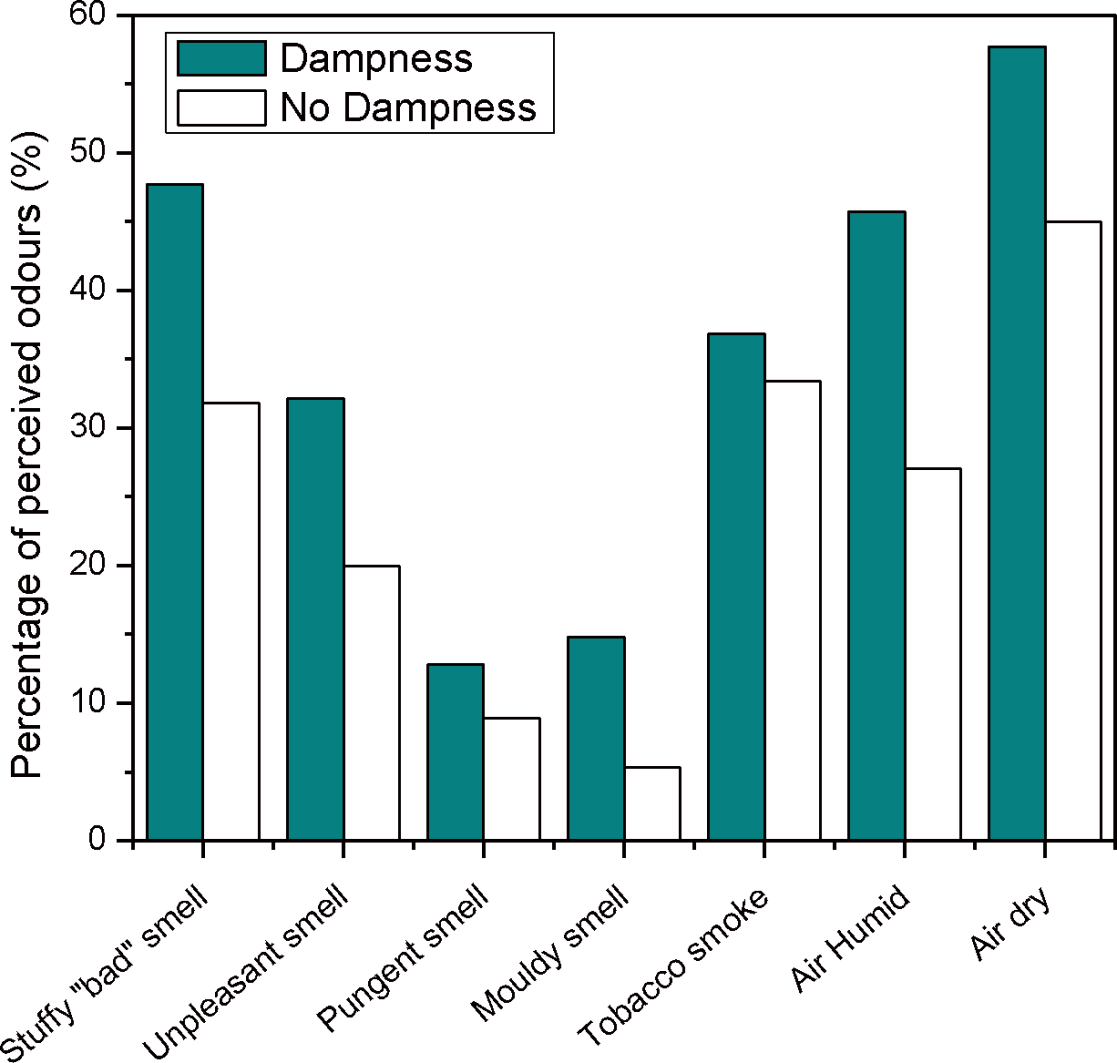
Percentages of homes with different odors, with and without dampness indices, as reported by the parents.

Dampness was defined as having at least one of four indicators: window condensation, visible mold, visible stain and bedding dampness. At least one indicator of dampness was reported for 61.5% of the children’s rooms. Of the 61.4% of homes reporting window condensation, 32.2% had condensation higher than 5 cm. About 26.2% of homes had damp bedding, but only 4.5% and 7.7% of homes found visible mold and visible damp stains respectively. Fig 1 compares odors in homes with and without dampness indicators. The proportions of perceived odors in dwellings with dampness were significantly higher than for dwellings without dampness (*P* < 0.001), except for tobacco smoke, *P* = 0.018). “Dry air” was the most commonly reported sensation. There were more reports of “dry air” in homes which had dampness indicators. All perceived odors except for tobacco smoke and except for perceived dampness were associated with dampness indices. In homes with window condensation, the sensation of dry air (55%) was reported more frequently than the sensation of humid air (40%). Detailed data are presented in S1 Table.

Older dwellings (before 1990) had more reported visible mold, visible damp stains and damp bedding than new dwellings (Table 3; *P* < 0.001). However, older dwellings were reported to have less window condensation than new dwellings (Table 3).

**Table 3:** The percentage of dampness indices for different construction year of the buildings.

		Before 1990	1991–2000	After 2001	*P*
Visible mold		8.5	4.8	2.8	< 0.001
Visible damp stain		13.6	9.4	4.6	< 0.001
Bedding damp		33.6	29.3	21.7	<0.001
Windows	>25cm	5.8	9.7	11.1	<0.001
condensation	5–25cm	12.4	16.2	16.9	
	<5cm	20.4	24.5	22.8	
	never	36.3	28.8	27.8	

Doctor-diagnosed asthma was reported for 8.7% of children. It was reported that 17.6% of children have had a dry cough at night not associated with a cold or chest infection for more than two weeks in last 12 months, and 17.9% of children were reported to have had wheeze or whistling in the chest in the last 12 months, while 27.1% of children had had eczema. Rhinitis was reported for 41.6% of the children, and lifetime-ever pneumonia for 26.7%. Prevalences of allergic diseases and pneumonia stratified by gender, age and family history are presented in S3 Table.

Chi-square tests were used to estimate crude associations between odors and humidity perceptions, and allergic diseases among children. Prevalences of children’s diseases in homes with and without odors are shown in Table 4. Prevalences of allergic diseases were higher in homes with reported odors.

**Table 4:** Number and prevalence of children allergic diseases, and pneumonia in homes with/without odors. (Confidence Intervals and Proportions in parentheses).

		Asthma	Wheeze	Eczema	Dry cough	Rhinitis symptom	Pneumonia
Stuffy odor	Yes	154(10.4)**	342(23.2)***	470(32.0)***	314(21.3)***	693(47.1)***	418(28.3)
	No	165(7.2)**	313(13.7)***	576(25.2)***	358(15.6)***	849(37.1)***	585(25.5)
Unpleasant odor	Yes	110(11.6)***	213(22.7)***	296(31.7)**	215(22.9)***	459(48.8)***	263(27.9)
	No	203(7.4)***	420(15.3)***	715(26.0)**	449(16.4)***	1047(38.2)***	710(25.8)
Pungent odor	Yes	48(12.1)**	96(24.4)***	134(34.1)**	94(23.7)**	193(49.1)***	127(32.1)**
	No	284(8.0)**	538(16.4)***	885(26.9)**	566(17.2)**	1315(39.9)***	852(25.8)**
Moldy odor	Yes	50(13.9)***	100(27.9)***	121(33.6)**	90(25.1)***	174(48.5)**	108(30.3)
	No	261(7.9)***	534(16.1)***	894(26.9)**	568(17.1)***	1332(40.1)**	869(26.1)
Tobacco odor	Yes	121(9.2)	251(19.3)*	385(29.5)	248(19.0)	596(45.6)***	367(28.0)
	No	193(7.9)	386(15.9)*	648(26.7)	417(17.2)	935(38.5)***	624(25.7)
Humid air	Yes	135(10.1)*	291(21.8)***	433(32.5)***	285(21.4)*	628(47.1)***	389(29.1)*
	No	185(7.7)*	354(14.7)***	605(25.1)***	382(15.8)*	908(37.7)***	608(25.2)*
Dry Air	Yes	191(10.1)***	378(20.1)***	585(31.0)***	396(21.0)***	861(45.7)***	541(28.6)**
	No	123(6.7)***	256(14.1)***	438(24.1)***	264(14.5)***	655(36.0)***	448(24.6)**

*P < 0.05

** *P* < 0.005

*** *P* < 0.001.

Table 5 shows odds ratios for children’s allergic diseases in relation to the perception of odors and dry air. Odds ratios were adjusted for children’s gender, age and family history. Perception of odors (except tobacco odors) and humidity were significantly associated with asthma and the allergic respiratory symptoms wheeze, dry cough and rhinitis (*P* < 0.05). Homes with tobacco odors were associated with wheeze, dry cough (*P* < 0.05) and rhinitis symptom (*P* < 0.001).

**Table 5:** Association between allergic diseases and perception of odors and humidity.

	Asthma	Wheeze	Eczema	Dry cough	Rhinitis	Pneumonia
Stuffy odor	1.36 (1.07–1.73)*	1.79 (1.50–2.14)***	1.29 (1.04–1.60)*	1.40 (1.18–1.66)***	1.45(1.27–1.67)***	1.09(0.93–1.26)
Unpleasant odor	1.53 (1.12–1.96)**	1.51 (1.24–1.83)***	1.16 (0.91–1.49)	1.48 (1.23–1.79)***	1.47 (1.26–1.72)***	1.06(0.89–1.26)
Pungent odor	1.43 (1.02–2.01)*	1.59 (1.20–2.02)**	1.20 (0.86–1.68)	1.52 (1.18–2.0)**	1.40 (1.13–1.74)**	1.32(1.05–1.66)*
Moldy odor	1.77 (1.26–2.48)**	1.91 (1.48–2.47)***	1.09 (0.76–1.55)	1.57 (1.21–2.04)**	1.37 (1.09–1.71)*	1.17(0.91–1.49)
Tobacco odor	1.22 (0.95–1.55)	1.27 (1.06–1.52)*	1.10 (0.88–1.38)	1.21 (1.01–1.45)*	1.36 (1.18–1.57)***	1.13(0.97–1.32)
Air humid	1.24 (0.97–1.58)	1.53 (1.28–1.83)***	1.20 (0.96–1.49)	1.35 (1.13–1.61)***	1.42 (1.24–1.64)***	1.16(0.99–1.36)
Air dry	1.40 (1.10–1.79)*	1.39 (1.16–1.66)***	1.21 (0.97–1.51)	1.51 (1.26–1.80)***	1.44 (1.26–1.65)***	1.17(0.99–1.35)

Adjusted for children’s gender, age and family member asthma or allergies history. Reference is the “no” group for perceived odors.

*P < 0.05

** *P* < 0.005

*** *P* < 0.001.

We built an odor and humidity perception index scale. The scale (O&H) is the sum of yes responses for odors (stuffy, unpleasant, pungent, moldy, tobacco), and humidity sensations (humid air, dry air), reported either ‘weekly’ or ‘sometimes’ given a score of one. For example, stuffy (weekly), plus moldy (sometimes), plus humid (sometimes), plus dry (weekly) would receive a score of 4.

Associations between children’s allergic diseases and the O&H score were calculated using logistic regression (see Table 6). All adjusted ORs except for pneumonia increased with increasing O&H score.

**Table 6:** Associations between children’s allergic diseases and O&H-score.

	Categorized O&H score	OR^a^ (95%CI)	*P*
Asthma	Score category 0	1.00	
	Score category 1	1.28(0.86–1.91)	0.23
	Score category 2	1.35(0.94–1.94)	0.10
	Score category 3	**1.81(1.23–2.67)**	0.003
Wheeze	Score category 0	1.00	
	Score category 1	**1.42(1.05–1.91)**	0.02
	Score category 2	**1.64(1.26–2.15)**	<0.001
	Score category 3	**2.58(1.94–3.43)**	<0.001
Eczema	Score category 0	1.00	
	Score category 1	0.90(0.63–1.30)	0.58
	Score category 2	1.33(0.98–1.80)	0.07
	Score category 3	1.25(0.88–1.77)	0.21
Dry cough	Score category 0	1.00	
	Score category 1	1.30(0.97–1.75)	0.07
	Score category 2	**1.83(1.41–2.36)**	<0.001
	Score category 3	**2.14(1.61–2.84)**	<0.001
Rhinitis	Score category 0	1.00	
	Score category 1	**1.27(1.03–1.57)**	0.03
	Score category 2	**1.69(1.40–2.04)**	<0.001
	Score category 3	**2.18(1.76–2.70)**	<0.001
Pneumonia	Score category 0	1.00	
	Score category 1	0.95(0.76–1.20)	0.67
	Score category 2	0.87(0.72–1.07)	0.18
	Score category 3	0.85(0.68–1.06)	0.15

^a^ Adjusted for children’s gender, age and family history.

## Discussion

Perceived odors (except tobacco odor) were found to be significantly associated with children’s respiratory allergic diseases including asthma, wheeze, dry cough and rhinitis symptoms. Engvall et al.[19] reported that odor perceptions were significantly associated with adults’ asthmatic symptoms with ORs (95%CI) as follows: pungent (OR 4.03; 95%CI 3.78–4.29), moldy (OR 3.24; 95%CI 3.07–3.41), and stuffy odors (OR 2.45; 95%CI 2.36–2.54). Another study in Sweden [26] found moldy odor to be associated with allergic symptoms, especially rhinitis, among children. They also observed that while the OR(95%CI) for high ventilation was 2.52(1.11–5.76), the OR(95%CI) for low ventilation was even greater, at 3.01(1.52–5.96).

We found that several environmental factors were associated with increased perceptions of odor or humidity: older, smaller homes, perceived dampness, and location in an industrial neighborhood. Residents of industrial areas more often perceived unpleasant, pungent and tobacco odors than residents of urban and suburban areas and were at the greatest risk for perception of pungent odors (*P* <0.001). Old buildings were reported to have more moisture related problems, namely moldy odor, visible mold, visible damp stain and damp bedding (Table 3). This finding may explain why older dwellings were reported to have more unpleasant, moldy and tobacco odors, as well as the sensation of humid air, than newer dwellings (Table 2). This finding is consistent with previous Swedish [27] and Korean studies [28]. It may be explained by poor maintenance and less insulation than in newer buildings. Complaints about odor and humidity were more common for smaller dwellings. To some extent, house size is a proxy for socio-economic status. Low-income families may be forced to live in smaller dwellings which lack good maintenance and insulation.

An interesting finding was that the proportion of “dry air” reports was significantly higher in homes where signs of dampness, especially condensation on windows were reported. Sundell and Lindvall [21] found that condensation on windows was associated with high indoor humidity and a low ventilation rate. There are many moisture sources indoors, including human respiration and any combustion process; a low ventilation rate allows water vapor as well as pollutants generated indoors to accumulate. Fang et al [29] found that the sensation of dryness was enhanced by reduced ventilation. Thus, our apparent paradoxical finding is consistent with what has been previously observed, and can be explained as the sensation of dry air being due to an increased level of pollutants rather than by actual dryness [18].

Perceived tobacco odor was significantly associated with children’s wheeze (OR 1.27; 95%CI 1.06–1.52), dry cough (OR 1.21; 95%CI 1.01–1.45) and rhinitis (OR 1.36; 95%CI 1.18–1.57). A number of studies [30–35] have reported that ETS (environmental tobacco smoking) exposure has significant adverse effects on respiratory health. A study in Taiwan [31] found that exposure to both parents’ smoking was significantly associated with early-onset asthma (OR 2.01; 95%CI 1.00–4.02). Tabuchi et al.[32] reported that the increased odds for children whose father and mother both smoke indoors was 54% (95% confidence interval: 21–96%) compared to children with non-smoking parents. Zuraimi’s study[33] concluded that home ETS exposure was associated with increased risk of current rhinitis (OR 1.23; 95% CI 1.01–1.50) and rhinoconjunctivitis (OR 1.79; 95% CI 1.26–2.54).

We found an association between tobacco odor perception and children’s respiratory diseases. However, we found no significant association between parental smoking and children’s allergic diseases (S4 Table). Although tobacco odor is strongly suggestive of ETS exposure, it is possible that in China, many parents avoid smoking in the presence of children indoors. The Chinese “one child one family” policy has strengthened parental consciousness of children’s health. Therefore, it is necessary to ask whether parents smoke in the presence of children to accurately estimate children’s ETS exposure in China.

Perceived odors are likely due to pollutants, especially mixtures of VOCs in indoor air at low concentrations. Thus, perceived odors may also reflect a low ventilation rate [36]. Environmental factors are thought to be associated with asthma and other respiratory allergic diseases [4,7,37]. The findings of the present study indicate that perceived odors are associated with a risky home environment for children’s respiratory diseases.

Epidemiological studies may be subject to selection bias. The sample size for this study was large, had a reasonable response rate (65.7%) and covered all districts of Nanjing except for two outlying counties. Thus, selection bias is reasonably unlikely. Recall bias is another potential problem, as subjects may overestimate or underestimate their child’s symptoms and/or signs of indoor environmental risk factors. Recall bias for odors and humidity perception, as well as children’s asthma or allergic disease symptoms should not be severe in this study, since the recall period was short. There is also risk of systematic over-reporting of indoor air problems for diseased subjects and/or a risk for over-reporting symptoms in subjects who live in problem buildings [38,39]. In this study, there are two potential reporting biases (S4 Table). First, there can be a bias for subjects who are aware that certain factors have previously been identified as risks. The other reporting bias is a gender difference; females report perceptions of odors and humidity more often than males [40]. This has been observed in previous studies [40] including a CCHH study of Chongqing [20]. To account for reporting bias, we have stratified for family history of asthma or allergies and gender of the questionnaire responder. We still found associations between odors and humidity perceptions, and children’s health outcomes, indicating that reporting bias did not significantly affect our findings (See S5 and S6 Tables).

## Conclusions

Perceptions of odors and sensation of humidity, as reported by parents, was positively associated with children’s diagnosed asthma and the occurrence of dry cough, wheeze or whistling in the chest, eczema and rhinitis symptoms. Homes located in industrial areas, older homes, and smaller homes were positively associated perceptions of odors. Homes located in industrial areas were found to be associated more with pungent odors. Smaller and older dwellings tended to have more moldy odor. The sensation of dryness was positively associated with dampness, suggesting that irritating substances in the air, rather than actual dryness, may have caused the “dryness” sensation. With the exception of tobacco odors, parents’ perceptions of all queried odors, and evidence of dampness were found to be significantly associated with respiratory allergic diseases (asthma, wheeze, dry cough and rhinitis). The results suggest that unpleasant smells in a dwelling indicate relatively unhealthful indoor air.

## Supporting Information

S1 File Questions about children's health, building characteristic and perceived air quality. (DOCX)

S1 Table Number and proportions of surveyed parents with perceived odors and sensations of humidity and dryness as related to dwelling floor areas. (DOCX)

S2 Table Number and proportions of surveyed homes with perceived odors, or the sensation of humid or dry related to dampness indicators. (DOCX)

S3 Table Association between children’s allergic diseases and parental reported smoking. (DOCX)

S4 Table Prevalence of children allergic diseases and pneumonia. (DOCX)

S5 Table Prevalence of children allergic diseases in homes with/without odors or the sensation humid or dry air, stratified for family history of asthma or allergies. (DOCX)

S6 Table Association between allergic diseases and perception of odors or the sensation of humidity or dryness, stratified for gender of responders to the questionnaire. (DOCX)
