# Supplementary material for: Associations between Parents’ Perceived Air Quality in Homes and Health among Children in Nanjing, China
Source: PLoS One. 2016 May 18;11(5):e0155742. doi: 10.1371/journal.pone.0155742 (PMC4871534; doi:10.1371/journal.pone.0155742)
Supplement: S1 File — (DOCX) [file pone.0155742.s001.docx]

S1 File: Questions about children's health, building characteristic and perceived air quality.

(1) Demographic information:

(a) Child’s Gender (male vs. female);

(b) Child’s Age;

(c) Who filled out the questionnaire (father, mother, grandfather, grandmother or others);

d) Has any members of your family had asthma or allergies?(yes vs.no)

(2) Health

1. Has a doctor ever diagnosed your child with asthma? (yes vs. no); (asthma)
2. Has your child ever had wheeze or whistling in the chest in the last 12 months? (yes vs. no);(wheeze)
3. Has your child ever had eczema in the recent past 12 months? (yes vs. no); (eczema)
4. In the last 12 months, has your child had a dry cough at night for more than two weeks, apart from a cough associated with a cold or chest infection? (yes vs. no); (dry cough)
5. Has a doctor ever diagnosed your child with pneumonia? (yes vs. no); (pneumonia)
6. In the past 12 months, has your child had a problem with sneezing, or a runny, or a blocked nose when he/ she did not have a cold or the flu? (yes vs. no); (rhinitis symptom)

(3) Building characteristics

(a) Indicate the general location of your home (urban area vs. suburban area vs. industrial area)

(b) Can you approximately estimate the size of your residence? ( <75 m2 vs. >75 m2)

(c) Can you state, approximately, the year that the residence was built? (Prior to 1990 vs 1990-2000 vs 2000- now)

(4) Dampness indicators

1. In the winter, does condensation or moisture occur on the inside, at the bottom, of window panes in the child’s room? (never vs. <5 cm vs. 5-25 cm vs. >25 cm);
2. Have you ever noticed any visible mold on the floor, walls or ceiling in your child’s room? (yes vs. no);
3. Have you ever noticed any visible damp stains on the floor, walls or ceiling in your child’s room? (yes vs. no);
4. Have you ever noticed your clothing and/or bedding being damp during the last year? (yes vs. no);

(5) Parents or another guardian’s perceived air quality

Have you during the last 3 months been bothered by any (one or more) of the odors, stated below, in your residence? Stuffy ‘bad’ smell; Unpleasant smell; Pungent smell; Moldy smell; Tobacco smoke; Humid air; Dry air; (yes, frequently (weekly) vs. yes, sometimes vs. no, never) [the data for yes frequently and yes sometimes were combined as yes in this paper].
