# Supplementary material for: Associations between Parents’ Perceived Air Quality in Homes and Health among Children in Nanjing, China
Source: PLoS One. 2016 May 18;11(5):e0155742. doi: 10.1371/journal.pone.0155742 (PMC4871534; doi:10.1371/journal.pone.0155742)
Supplement: S1 Table — (DOCX) [file pone.0155742.s002.docx]

S1 Table: Number and proportions of surveyed parents with various odor and humidity perceptions as related to the floor areas of their homes (Percentage in brackets)

|  | < 75 m2 | | ≥ 75 m2 | | P* |
| --- | --- | --- | --- | --- | --- |
|  | Weekly | Sometime | Weekly | Sometime |  |
| Stuffy odor | 44 (2.7) | 606 (37.7) | 54 (2.5) | 765 (35.6) | 0.359 |
| Unpleasant odor | 22 (1.4) | 420 (26.7) | 24 (1.1) | 473 (22.5) | **0.008** |
| Pungent odor | 9 (0.6) | 176 (11.2) | 8 (0.4) | 198 (9.4) | 0.123 |
| Moldy odor | 12 (0.8) | 180 (11.5) | 4 (0.2) | 164 (7.8) | **<0.001** |
| Tobacco odor | 115 (7.2) | 444 (27.8) | 111 (5.2) | 627 (29.4) | **0.033** |
| Humid air | 22 (1.4) | 578 (36.1) | 18 (0.8) | 711 (33.3) | **0.046** |
| Dry air | 49 (3.1) | 764 (48.2) | 66 (3.1) | 1006 (47.5) | 0.931 |
