# Supplementary material for: Associations between Parents’ Perceived Air Quality in Homes and Health among Children in Nanjing, China
Source: PLoS One. 2016 May 18;11(5):e0155742. doi: 10.1371/journal.pone.0155742 (PMC4871534; doi:10.1371/journal.pone.0155742)
Supplement: S2 Table — (DOCX) [file pone.0155742.s003.docx]

S2 Table: Number and proportions of surveyed homes with perceived odors, or the sensation of humid or dry related to dampness indicators

| **Dampness indices** |  | Stuff "bad" smell | Unpleasant smell | Pungent smell | Moldy smell | Tobacco smoke | Sensation of Humidity | Sensation of Dry Air |
| --- | --- | --- | --- | --- | --- | --- | --- | --- |
| Visible mold | Yes | 93(56.0)*** | 57(36.5)** | 28(17.7)** | 57(36.5)*** | 63(39.4) | 93 (57.8)*** | 98 (62.0)** |
|  | No | 1290(37.5)*** | 827(24.5)** | 347(10.3)** | 276(8.2)*** | 1184(34.6) | 1175(34.2)*** | 1712 (50.3)** |
| Visible damp stain | Yes | 153(55.4)*** | 110(41.5)*** | 54(19.9)*** | 84(30.9)*** | 100(36.9) | 180 ( 65.4)*** | 170 (63.2)*** |
|  | No | 1258(37.3)*** | 791(23.9)*** | 322(9.7)*** | 259(7.8)*** | 1161(34.6) | 1102(32.8)*** | 1657(49.8)*** |
| Bedding damp | Yes | 527(54.0)*** | 359(38.2)*** | 163(17.2)*** | 216(22.8)*** | 399(41.5)*** | 556 (57.0)*** | 583 (61.2)*** |
|  | No | 950(34.0)*** | 588(21.4)*** | 232(8.5)*** | 145(5.3)*** | 911(32.8)*** | 787(28.4)*** | 1312(47.5)*** |
| Windows condensation | >25cm | 176(48.4)*** | 112(31.8)*** | 47(13.2)** | 43(12.1)* | 115(31.7)* | 141 (39.2)*** | 200 (55.6)*** |
|  | 5-25cm | 249(42.2)*** | 152(26.3)*** | 45(7.8)** | 49(8.5)* | 200(34.3)* | 225(38.3)*** | 322 (55.5)*** |
|  | <5cm | 386(45.8)*** | 250(30.2)*** | 116(14)** | 101(12.2)* | 322(38.3)* | 352(41.7)*** | 461 (55.2)*** |
|  | 0 | 329(29.3)*** | 220(20)*** | 112(10.1)** | 95(8.6)* | 354(31.6)* | 333(29.6)*** | 471 (42.3)*** |

*** *P* < 0.001, ** *P* < 0.005, * *P* < 0.05.

Except for window condensation, the *P* value in the table was calculated using a 2*2 contingency table and Pearson χ^2^ test. Window condensation was calculated using a 2*4 contingency table and Pearson χ^2^ test.
