# Supplementary material for: Associations between Parents’ Perceived Air Quality in Homes and Health among Children in Nanjing, China
Source: PLoS One. 2016 May 18;11(5):e0155742. doi: 10.1371/journal.pone.0155742 (PMC4871534; doi:10.1371/journal.pone.0155742)
Supplement: S3 Table — (DOCX) [file pone.0155742.s004.docx]

S3 Table: Association between children’s allergic diseases and parental reported smoking

|  | Asthma | Wheeze | Eczema | Dry cough | Rhinitis |
| --- | --- | --- | --- | --- | --- |
| Family member smoking | 1.03 (0.82-1.30) | 0.96 (0.81-1.14) | 0.88 (0.71-1.08) | 0.91(0.77-1.08) | 0.91(0.79-1.04) |
| Parental smoking during the first year of child’s life | 1.10 (0.88-1.38) | 1.00 (0.85-1.18) | 0.93 (0.75-1.14) | 0.98 (0.83-1.16) | 0.90 (0.79-1.02) |
| Parental smoking during pregnancy | 1.09 (0.87-1.36) | 0.99 (0.85-1.18) | 0.93 (0.75-1.14) | 0.97 (0.82-1.15) | 0.89(0.78-1.01) |

Adjusted for children’s gender, age and family member asthma or allergies history.

”no” group of perceived odors was reference;
