# Supplementary material for: Associations between Parents’ Perceived Air Quality in Homes and Health among Children in Nanjing, China
Source: PLoS One. 2016 May 18;11(5):e0155742. doi: 10.1371/journal.pone.0155742 (PMC4871534; doi:10.1371/journal.pone.0155742)
Supplement: S4 Table — (DOCX) [file pone.0155742.s005.docx]

S4 Table: Prevalence of children’s allergic diseases and pneumonia

|  |  | **Gender** | | **Age** | | **History of asthma or allergies in family** | |
| --- | --- | --- | --- | --- | --- | --- | --- |
|  | Total | male | female | ≤5 years old | >5 years old | no | yes |
| **Asthma** | 8.7 | 10.9*** | 6.3*** | 8.8 | 8.8 | 6.5*** | 20.3*** |
| **Wheeze** | 17.8 | 19.9** | 15.7** | 18.8* | 16.2* | 15.6*** | 28.8*** |
| **Eczema** | 10 | 11* | 9* | 11.2** | 8.4** | 8.6*** | 17.9*** |
| **Dry cough** | 17.6 | 18.3 | 16.9 | 20.2*** | 13.6*** | 15.3*** | 30.6*** |
| **Rhinitis symptom** | 41.6 | 44.2** | 38.9** | 43.5* | 39.2* | 38.3*** | 59.6*** |
| **Pneumonia** | 26.7 | 28.8** | 24.5** | 26.9 | 26.4 | 24.9*** | 35.7*** |

**P* < 0.05, ***P* < 0.005, ****P* < 0.005
