# Supplementary material for: Associations between Parents’ Perceived Air Quality in Homes and Health among Children in Nanjing, China
Source: PLoS One. 2016 May 18;11(5):e0155742. doi: 10.1371/journal.pone.0155742 (PMC4871534; doi:10.1371/journal.pone.0155742)
Supplement: S5 Table — (DOCX) [file pone.0155742.s006.docx]

S5 Table: Prevalence of children’s allergic diseases in homes with/without odors, or the sensation of humid or dry air, stratified for a family history of asthma or allergies.

| **No ^a^** | | **Asthma** | **Wheeze** | **Eczema** | **Dry cough** | **Rhinitis symptom** |
| --- | --- | --- | --- | --- | --- | --- |
| **Stuffy odor** | Yes | 7.1 | 20.6*** | 10.4* | 17.6* | 42.2*** |
|  | No | 5.8 | 12.2*** | 7.8** | 14.3* | 34.9*** |
| **Unpleasant odor** | Yes | 8.3* | 19.9*** | 9.2* | 19.4*** | 44.0*** |
|  | No | 5.6* | 13.6*** | 8.4* | 14.5*** | 35.5*** |
| **Pungent odor** | Yes | 9.7* | 20.9** | 10.1 | 19.4 | 44.6* |
|  | No | 6.0* | 14.5** | 8.5 | 15.1 | 36.8** |
| **Moldy odor** | Yes | 10.2** | 23.3*** | 8.8 | 23.7*** | 41.9 |
|  | No | 5.9** | 14.4*** | 8.6 | 14.8*** | 37.1 |
| **Tobacco odor** | Yes | 7.3 | 17.6** | 8.8 | 16.9 | 43.1*** |
|  | No | 5.9 | 13.8*** | 8.6 | 14.8 | 34.8*** |
| **Air humid** | Yes | 7.5 | 19.5*** | 9.4 | 18.2** | 43.5*** |
|  | No | 5.8 | 12.9*** | 8.2 | 14.2** | 34.8*** |
| **Air dry** | Yes | 7.5** | 17.4*** | 9.5 | 18.0*** | 41.5*** |
|  | No | 6.7** | 12.6*** | 7.6 | 12.8*** | 33.5*** |
| **Yes^a^** | | **Asthma** | **Wheeze** | **Eczema** | **Dry cough** | **Rhinitis symptom** |
| **Stuffy odor** | Yes | 23.9* | 33.0** | 18.5 | 37.3** | 67.3*** |
|  | No | 16.6* | 22.6** | 17.3 | 25.1** | 51.5*** |
| **Unpleasant odor** | Yes | 24.6 | 31.3 | 18.9 | 38.3* | 67.4** |
|  | No | 17.9 | 25.4 | 16.0 | 28.2* | 54.9** |
| **Pungent odor** | Yes | 20.7 | 35.8 | 17.1 | 42.5* | 65.4 |
|  | No | 19.6 | 26.2 | 17.4 | 29.5*s | 58.1 |
| **Moldy odor** | Yes | 27.8 | 44.4** | 19.4 | 32.4 | 73.6* |
|  | No | 18.8 | 24.9** | 17.2 | 30.8 | 67.3* |
| **Tobacco odor** | Yes | 18.8 | 28.0 | 18.9 | 32.3 | 57.7*** |
|  | No | 20.4 | 26.4 | 16.8 | 30.0 | 59.3*** |
| **Air humid** | Yes | 21.1 | 30.5 | 19.5 | 35.0 | 61.2 |
|  | No | 19.0 | 25.1 | 15.8 | 27.9 | 57.3 |
| **Air dry** | Yes | 21.8 | 31.0* | 18.0 | 35.8*** | 64.8** |
|  | No | 18.3 | 23.4* | 16.3 | 25*** | 52.4** |

^a^ Family history of asthma or allergies (yes or no)

**P* < 0.05, ***P* < 0.005, ****P* < 0.005
