# Supplementary material for: Associations between Parents’ Perceived Air Quality in Homes and Health among Children in Nanjing, China
Source: PLoS One. 2016 May 18;11(5):e0155742. doi: 10.1371/journal.pone.0155742 (PMC4871534; doi:10.1371/journal.pone.0155742)
Supplement: S6 Table — (DOCX) [file pone.0155742.s007.docx]

S6 Table: Association between allergic diseases and perception of odors or the sensation of humidity or dryness, stratified for gender of responders to the questionnaire.

|  | | **Asthma** | **Wheeze** | **Eczema** | **Dry cough** | **Rhinitis symptom** |
| --- | --- | --- | --- | --- | --- | --- |
| **Stuffy odor** | male^b^ | 1.73(1.10-2.72)** | 2.04(1.42-2.93)*** | 1.30(0.82-2.05) | 20.9*1.56(1.09-2.23)* | 1.63(1.22-2.16)*** |
|  | female^b^ | 1.25(0.94-1.66) | 1.68(1.37-2.06)*** | 1.25(0.97-1.61) | 1.31(1.07-1.60)* | 1.39(1.19-1.64)*** |
| **Unpleasant odor** | male^b^ | 1.75(1.07-2.89)* | 1.89(1.26-2.83)** | 1.65(1.00-2,73)* | 2.20(1.50-3.23)*** | 1.63(1.17-2.25)** |
|  | female^b^ | 1.48(1.10-2.70)* | 1.384(1.11-1.73)** | 1.04(0.78-1.39) | 1.27(1.01-1.58)* | 1.42(1.19-1.70)*** |
| **Pungent odor** | male^b^ | 1.77(0.91-3.48) | 1.25(0.68-2.27) | 1.45(0.71-2.98) | 1.08(0.60-1.97) | 1.65(1.04-2.61)* |
|  | female^b^ | 1.38(0.93-2.05) | 1.55(1.16-2.08)** | 1.16(0.79-1.71) | 1.54(1.15-2.07)** | 1.31(1.02-1.69)* |
| **Moldy odor** | male^b^ | 2.50(1.39-4.49)** | 1.70(1.01-2.86)* | 1.27(0.64-2.51) | 1.50(0.89-2.52) | 1.75(1.13-2.71)* |
|  | female^b^ | 1.45(0.95-2.22) | 2.05(1.52-2.78)*** | 1.07(0.70-1.63) | 1.59(1.16-2.170** | 1.24(0.95-1.63) |
| **Tobacco odor** | male^b^ | 0.96(0.59-1.54) | 1.11(0.76-1.63) | 0.89(0.55-1.44) | 1.06(0.73-1.53) | 1.35(1.01-1.79)* |
|  | female^b^ | 1.35(1.013-1.80)* | 1.34(1.09-1.66)* | 1.13(0.87-1.48) | 1.269(1.02-1.56)* | 1.35(1.14-1.60)*** |
| **Air** | male^b^ | 1.38(0.87-2.19) | 1.34(0.93-1.94) | 1.19(0.74-1.89) | 1.76(1.24-2.51)** | 1.86(1.40-2.46)*** |
| **humid** | female^b^ | 1.20(0.90-1.59) | 1.56(1.27-1.92)*** | 1.17(0.91-1.52) | 1.18(0.96-1.45) | 1.32(1.12-1.55)** |
| **Air dry** | male^b^ | 1.49(1.00-2.37)* | 1.33(1.02-1.90)* | 1.28(0.81-2.00) | 1.34(0.94-1.9) | 1.45(1.11-1.89)*** |
|  | female^b^ | 1.36(1.01-1.82)* | 1.43(1.36-1.77)** | 1.18(0.91-1.51) | 1.50(1.22-1.85)*** | 1.43(1.21-1.68)*** |

^b^Gender of people who completed the questionnaire

OR(95%CI) adjusted for children’s gender, age and family history of asthma or allergies. Reference: “no” group of perceived odors

**P* < 0.05, ***P* < 0.005, ****P* < 0.001.
